# Temporal evolution of mechanical stimuli from vascular remodeling in response to the severity and duration of aortic coarctation in a preclinical model

**DOI:** 10.1038/s41598-023-34400-8

**Published:** 2023-05-23

**Authors:** Jamasp Azarnoosh, Arash Ghorbannia, El-Sayed H. Ibrahim, Hilda Jurkiewicz, Lindsey Kalvin, John F. LaDisa

**Affiliations:** 1 Department of Pediatrics - Section of Cardiology, Medical College of Wisconsin, Milwaukee, WI USA; 2 Department of Biomedical Engineering, Marquette University and the Medical College of Wisconsin, Milwaukee, WI USA; 3 Departments of Radiology, Medical College of Wisconsin, Milwaukee, WI USA; 4 Departments of Medicine - Division of Cardiovascular Medicine, Medical College of Wisconsin, Milwaukee, WI USA; 5 Departments of Physiology, Medical College of Wisconsin, Milwaukee, WI USA; 6 Herma Heart Institute, Children’s Wisconsin, Milwaukee, WI USA

**Keywords:** Congenital heart defects, Biomedical engineering

## Abstract

Coarctation of the aorta (CoA) is one of the most common congenital cardiovascular diseases. CoA patients frequently undergo surgical repair, but hypertension (HTN) is still common. The current treatment guideline has revealed irreversible changes in structure and function, yet revised severity guidelines have not been proposed. Our objective was to quantify temporal alterations in mechanical stimuli and changes in arterial geometry in response to the range of CoA severities and durations (i.e. age of treatment) seen clinically. Rabbits were exposed to CoA resulting in peak-to-peak blood pressure gradient (BPG_pp_) severities of ≤ 10, 10–20, and ≥ 20 mmHg for a duration of ~ 1, 3, or 20 weeks using permanent, dissolvable, and rapidly dissolvable sutures. Elastic moduli and thickness were estimated from imaging and longitudinal fluid–structure interaction (FSI) simulations were conducted at different ages using geometries and boundary conditions from experimentally measured data. Mechanical stimuli were characterized including blood flow velocity patterns, wall tension, and radial strain. Experimental results show vascular alternations including thickening and stiffening proximal to the coarctation with increasing severity and/or duration of CoA. FSI simulations indicate wall tension in the proximal region increases markedly with coarctation severity. Importantly, even mild CoA induced stimuli for remodeling that exceeds values seen in adulthood if not treated early and using a BPG_pp_ lower than the current clinical threshold. The findings are aligned with observations from other species and provide some guidance for the values of mechanical stimuli that could be used to predict the likelihood of HTN in human patients with CoA.

## Introduction

Coarctation of the aorta (CoA) is one of the most common congenital cardiovascular diseases. CoA often presents at birth as a narrowing that is most frequently treated surgically^1,2^. The current treatment guideline references a peak-to-peak blood pressure gradient (BPG_pp_) ≥ 20 mmHg^3–5^ despite implementation revealing irreversible changes in arterial structure and function in preclinical models^6^. For example, mechanical stimuli imposed by untreated CoA can lead to elevated pulse blood pressure (BP) proximal to the coarctation with adverse arterial remodeling^6^. Treated (i.e. corrected) CoA patients have also shown persistent structural and functional changes in the proximal carotid arteries.^7,8^ Importantly, many CoA patients have long-term morbidity in the form of refractory hypertension (HTN) even after successful surgical treatment^9^.

The findings above suggest the temporal mechanical stimuli contributing to persistent HTN in CoA are not well understood, which is the focus of our research. Previous studies of pathologic aortic remodeling have been limited to older ages^1,9–11^, but knowledge of mechanical stimuli at younger ages that could avoid permanent vascular remodeling is missing. The objective of this study was to quantify, for the first time, temporal alterations in mechanical stimuli and changes in arterial geometry in response to the range of CoA severities and durations seen clinically. A novel experimental rabbit model of CoA was designed with a protocol developed to create computational geometries for fluid–structure interaction (FSI) simulations that link longitudinal imaging to mechanical stimuli including blood flow velocity patterns, wall tension, and radial strain.

## Methods

### Experimental protocol

This study is reported in accordance with ARRIVE guidelines. All methods were also performed in accordance with the relevant ethical guidelines and regulations. Approval for the study was obtained from the Institutional Animal Care and Use Committee (IACUC). New Zealand White rabbits (74; n = 5–11/group), obtained from Kuiper Rabbit Ranch (Gary, IN), were randomly selected for exposure to BPG_pp_ severities ≤ 10, 10–20, or ≥ 20 mmHg for durations of ~ 1, 3, or 20 weeks by extending the model of Menon et al.^10^. Duration of CoA in the current study refers to the time when coarctation is induced via suture until treatment via suture resorption and is meant to mimic the time in humans when the CoA manifests within the first week of life after the closing of the ductus arteriosus until treatment is implemented. The putative treatment guideline implemented for humans with CoA^3–5^ was assumed to be applicable in rabbits since BP is similar between species^12^. The order of treatments, measurements and room locations were consistently implemented throughout the study. Group allocations were completed prior to authoring the current work. Briefly, rabbits were anesthetized with ketamine and xylazine (22 and 2.5 mg/kg IM, respectively), underwent endotracheal intubation, and were maintained by ventilation with 1–2% isoflurane. The duration of the coarctation was varied using permanent silk (CoA), dissolvable Vicryl (dCoA), or rapidly dissolvable Vicryl (RdCoA) sutures tied around the aorta at the location CoA most often presents clinically^6,10^. The severity of CoA was varied by tying the suture around the aorta against a wire of known diameter, i.e., 2.6, 2.0, or 1.6 mm, resulting in group-wise BPG_pp_ severities of ≤ 10, 10–20, or ≥ 20 mmHg, respectively. Untreated (i.e. native) coarctation was replicated in the CoA groups, while the treated condition (i.e. corrected) was replicated via resorption of the Vicryl or Vicryl Rapid suture (Ethicon) used to create the coarctation in the dCoA and RdCoA groups, respectively. A nonexperimental group was also established as a control.

### Magnetic resonance imaging (MRI)

Anatomic and phase contrast MRI were conducted as described in detail elsewhere^6,10^ to create computational geometries and quantify blood flow for FSI simulations. Following administration of anesthesia, a saline-filled angiocath IV was inserted into the marginal ear vein for contrast-enhanced visualization of the thoracic aorta and its branches. Cardiovascular MRI was conducted with a 3 T GE Sigma Excite scanner (GE Healthcare, Waukesha, WI) using a quadrature knee coil and sequences described elsewhere^10,13^. Briefly, gadolinium (Gd; Omniscan gadodiamide; GE Healthcare, Princeton, NJ) was loaded into the distal end of extension tubing connected to the angiocath IV before being transiently injected at a rate of ~ 1.2 ml/s for contrast-enhanced MR angiography (MRA). Time-resolved cardiac-gated, 2D phase contrast-MRI (PC-MRI) data were then obtained orthogonal to the ascending aorta to quantify inlet flow and cardiac output (CO). PC-MRI data were also obtained for the innominate, left common carotid, and right common carotid arteries, as well as the level of the coarctation, descending, and distal aorta to determine the distribution of flow to aortic branches.

### Flow quantification

Flow was quantified for each rabbit in a semi-automated manner (Segment version 3.3 R9405d, Medviso AB, Lund, Sweden). Regions of interest were manually determined at the first time step of PC-MRI images before Segment automatically obtained the regions of interest in other time steps using a threshold-based method. Nearby muscle^14^ served as stationary tissue for second-order compensation. The method excludes all moving structures such as the heart, lungs, large arteries, and veins when determining errors from eddy currents and magnetic field inhomogeneities^15,16^. CO was calculated by integrating the area under the ascending aorta PC-MRI waveform^17^. Considering sagittal symmetry for subclavian arteries, the right and left subclavian artery flows were estimated by subtracting the right carotid flow from that of the innominate artery. Partial volume effects were expected in branches due to decreased special resolution relative to the ascending aorta^18^. To achieve mass conservation, any difference in inflow relative to the summed outflows was redistributed proportionate to outlet diameter^19,20^. Flow distributions were assumed to be consistent at younger ages.

### Blood pressure

Arterial catheterization was performed just prior to tissue harvest. Rabbits were anesthetized and BP waveforms were recorded simultaneously via fluid-filled catheters advanced to the ascending and abdominal aorta from the right carotid and femoral arteries, respectively^10^. The catheters (ID = 0.86 mm OD = 1.27 mm) were connected to Harvard Apparatus pressure transducers (model number APT300; Holliston, MA). Consistent with prior work, catheter-based measurements revealed mean BP remained near control levels despite coarctation severity^21^, likely due to the presence of collateral vessels^22–24^. Hence, a consistent mean BP was assumed temporally. Conversely, CoA can have a marked impact on pulse pressure (PP)^25^. Thus, PP was applied to FSI simulations at younger ages as a function of coarctation severity using the measured catheterization data.

Catheterization is invasive and was not available longitudinally. Hence, Doppler signal assessment by ultrasound (i.e. non-invasive) was used for velocity and BPG estimation at younger ages^26,27^. Ultrasound studies were performed by a trained sonographer to track evolution of the BPG as previously described in detail^13^. Briefly, transthoracic ultrasound was performed with an 11-MHz M12L linear array transducer interfaced to a Vivid 7 ultrasound system (GE Healthcare, Waukesha, WI). Closed chest imaging took place after the chest was carefully shaved to remove hair and allow for a gel interface between the skin and transducer. Long axis 2-D, color Doppler and pulsed spectral Doppler images were obtained from the area above, within, and below the coarctation region. The degree of angle correction was recorded for each animal, and this angle was repeated for all subsequent exams. Rabbits were imaged weekly until the BPG generally equilibrated for a given rabbit. Each examination took less than 10 min, and rabbits were monitored continuously until ambulatory and sternal after each examination. Peak velocity was measured using electronic calipers available on the ultrasound system. Three cardiac cycles were measured and the average used for reporting. BPG_pp_ was estimated using the simplified Bernoulli equation.

Doppler-based assessment is known to overestimate BPG due to factors from the simplified Bernoulli’s equation^27^ To mitigate these issues, a new Doppler-based index (transfer function) developed in our lab was used for improved estimation of a catheter pressure gradient. This allowed for the transfer of peak instantaneous Doppler gradient (PIDG) to corresponding catheter-based peak-instantaneous pressure gradient (PIBPG) i.e. PIDG = 1.76 PIBPG – 6.9^21^ based on data across a range of coarctation severities seen clinically. PIBPG for FSI simulations at younger ages was therefore calculated from weekly ultrasound data. Heart rates were also obtained from longitudinal ultrasounds and used to create a function based on rabbit age using a power-law regression model.

### Wall thickness

Arterial wall thickness was measured proximal and distal to the coarctation from longitudinal ultrasound images using ImageJ (imagej.net). A power-law regression model fit was again used to assess temporal changes in wall thickness at these locations for use with FSI simulations. Reliability of measurements was investigated using intraclass correlation coefficient (ICC) through SPSS software for metrics including thickness measurements of the aorta wall. Two observers obtained quantities for a random subgroup of samples (n = 10).

### Material Testing

After harvest, the ascending aorta and its branches from the arch were dissected and underwent material characterization using uniaxial extension testing (MTS Criterion Load Frame, MTS, Minneapolis, U.S.A.) at 37 ^O^C in an environmental chamber (MTS Bionix EnviroBath, Minneapolis, U.S.A.). Tissues were dissected with a length-to-width ratio of ~ 2.6 and preconditioned by stretching to 10% of the gauge length. Extension testing was performed at 10 mm/min until hyperelastic behavior was observed.

### Computational model creation

**Figure 1:**
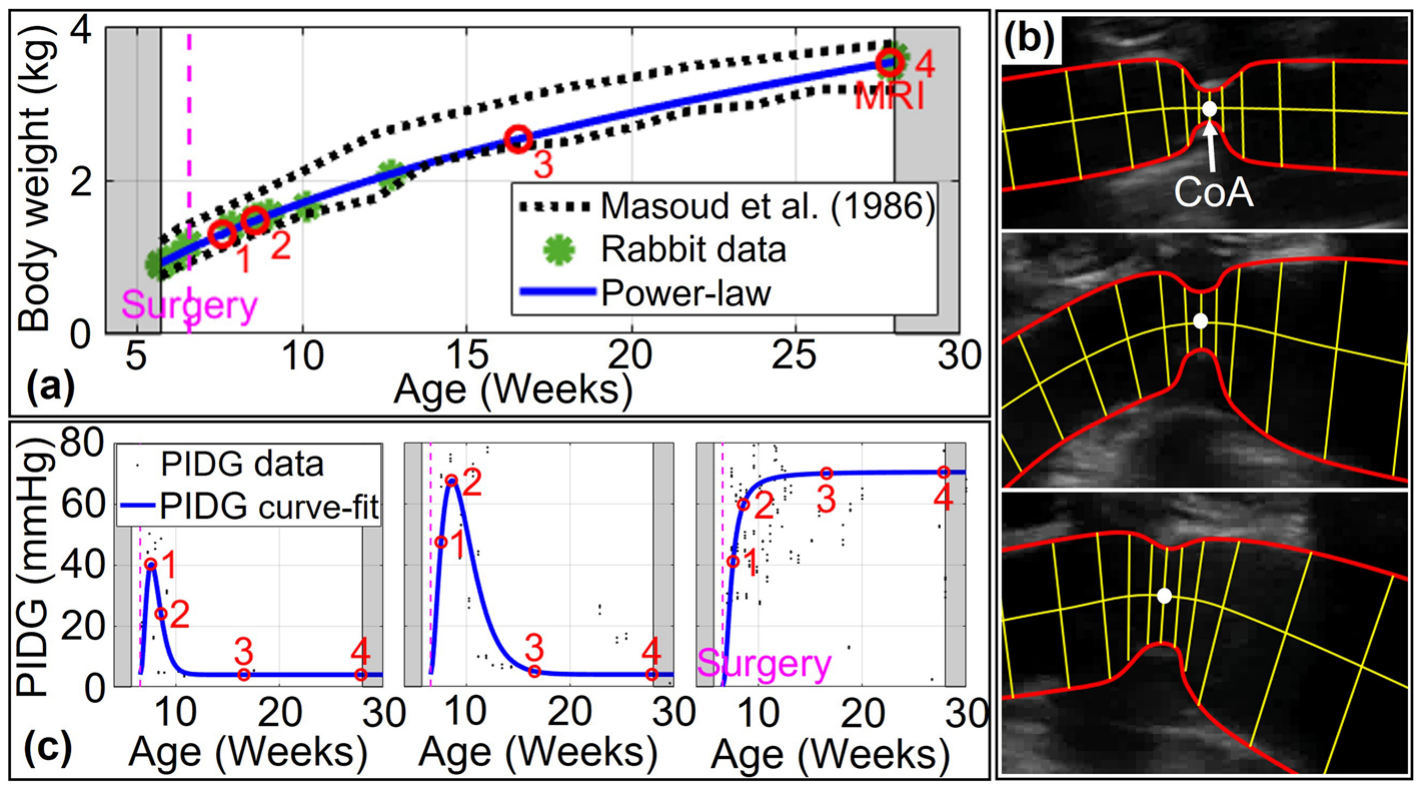
General approach used to create computational geometries. (**a**) MRI data are adjusted based on body size using allometric scaling to replicate geometries at younger ages (e.g. body weight vs. age for the dissolvable 20 mmHg BPG_pp_ rabbit at four time points). (**b**) A series of diastolic ultrasound images in a representative rabbit. (**c**) Temporal peak instantaneous Doppler pressure gradient (PIDG) for RdCoA, dCoA, and CoA from left to right, respectively.

It was intractable to create subject-specific models for each rabbit within the experimental groups and all for all time points studied (n = 74 rabbits yielding ~ 300 possible models and simulations). Due to time and resource constraints, only one rabbit per group was used for the group-based FSI simulation. Imaging data from the rabbit with blood pressure closest to the group average was used to create a representative model for each experimental group. The general approach to create computational geometries is shown in Fig. 1. Body weight was obtained during the experimental duration, which aligns with data from Masoud et al.^11^ (Fig. 1a). Data were fit to the allometric (power-law) equation, which is wildly used for scaling in biology^28,29^, to estimate geometries at younger ages. Subject-specific computational geometries representative of varying CoA severity and duration in each group were reconstructed using SimVascular (simvascular.github.io) from 3D MRI at the final week before tissue harvest (MRI week marked time point 4 in Fig. 1a). The model created for the final week was then leveraged to create geometries at younger ages using ultrasound images from the proximal to distal aorta that included the coarctation. Diameters were measured during diastole using ImageJ. Figure 1b shows segmentations assigned perpendicular to the centerline to measure the temporal evolution in the shape of the coarctation region. The length of each segment was measured relative to the coarctation. Diameters and longitudinal length measurements were imported into MATLAB to obtain the shape of the aorta by interpolation. The allometric equation was then used again to determine temporal changes in longitudinal diameters from proximal to distal locations. A similar procedure was performed during systole to determine arterial wall deformation i.e. percentage radial strain. Elastic modulus was determined by linearizing the hyperelastic stress–strain curves at the radial strain to be used in FSI simulations.

**Figure 2:**
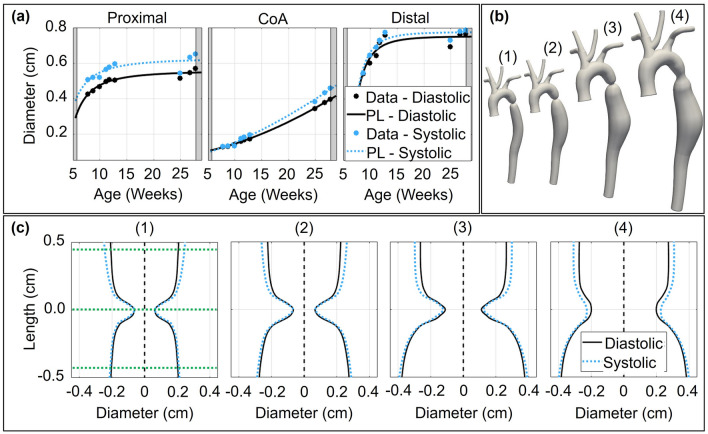
Temporal changes in CoA shape at four time points for dissolvable CoA with a severity of ≥ 20 mmHg. (**a**) Diastolic and systolic diameters with age at proximal, CoA, and distal locations were measured and fit to the power-law model. (**b**) The 3D geometries at four selected time points. (**c**) Temporal evolution of the CoA shape was measured using longitudinal ultrasound images (e.g. dCoA ≥ 20 mmHg BPG_pp_ at four time points) and incorporated into computational models. *PL* power-law model fit.

The CoA shape was determined at four time points (TP) of interest based on the approach to longitudinal ultrasound images described above. Time points at younger ages were selected based on PIDG in each group by Doppler-based estimation. The first time point represents the age when PIDG is observed in the rapid dissolvable group i.e. ~ 1 week after the surgery date (TP-1 in Fig. 1c). Similarly, the second time point is ~ 3 weeks after the surgery date when PIDG occurs in the dissolvable group (TP-2 in Fig. 1c). The third time point is the middle age of the rabbits (i.e. ~ 10 weeks after the surgery date) to evaluate the evolution of mechanical stimuli at this age from early weeks (TP-3 in Fig. 1c). TP-4 is the date when MRI is performed. The temporal evolution of the CoA shape is determined at the selected time points and integrated into 3D computational geometries to perform FSI simulations at each time point. For instance, Fig. 2c depicts temporal changes in the CoA shape at four time points for a dissolvable CoA with severity ≥ 20 mmHg.

### FSI simulations

In total, 40 simulations were conducted to account for the four time points studied in each group. Computational models were discretized using an automatic mesh generation program (TetGen; Berlin). A radius-based meshing technique with boundary layer meshing (5 layers) was used to locally increase the mesh density in locations with complex flow patterns^30^. A mesh convergence study was performed on the most severe case (permanent CoA ≥ 20 mmHg) where jet flow appears with a high velocity gradient due to the coarctation.

Time-dependent simulations were conducted using the finite element method within SimVascular to solve the conservation of mass, balance of fluid momentum, and Hooks equation governing arterial wall deformation in a coupled momentum method^31^. Elastic modulus was assigned to model locations based on experimental measurements within the physiological range of stress–strain hyperelastic curves. Elastic modulus at younger ages was assessed in the simulation tuning process based on radial strain from systolic and diastolic diameters in ultrasound imaging measurements. Blood was assumed as a Newtonian fluid with 1.06 g/cm^3^ and a viscosity of 4 cP consistent with a previous report in rabbits^6,10,32^. A no-slip condition was assumed along the wall.

### Boundary conditions

Ascending aortic flow waveforms were applied at the inlet face as a parabolic velocity profile. Waveforms imposed were similar for all time points, but the magnitude was scaled based on body weight consistent with reports showing a proportional relationship with CO^33,34^. Hence, the CO obtained at the MRI date (TP-4) was scaled down using the power-law equation fit to body weight data.

The total resistance for each outlet was calculated from the ratio of BP and PC-MRI flow distributions. Three-element Windkessel approximations were used to replicate physiology downstream from model outlets^28^ using parameters (see Supplemental Materials for all parameters) for the characteristic resistance (R_c_), capacitance (C), and distal resistance (R_d_). Estimation of these parameters used time constants ($\tau$) determined from the measured catheter BP waveforms proximal and distal to the coarctation. $\tau$ was defined as the exponential decay of diastolic pressure when flow is zero^28^, which was given by the product of R_d_ and C i.e. $\tau$ = R_d_C. Decay from the analytical solution governed by aortic BP is described by C = − t/R_d_ log(P/P_o_)^35^. To calculate C, the time, t, from an early diastolic pressure (P_o_) at the valve closure to end diastolic pressure (P) was considered. R_c_/R_tot_ was then iteratively adjusted using reported ranges^36^ to achieve measured flow and BP at outlet branches for each time point.

External tissue support was prescribed to limit non-physiological high-frequency oscillations of models using spring and damping constants of 10^3^ g/(cm^2^ s^2^) and 10^4^ g/(cm^2^ s), respectively.^37,38^ Simulations were run for five cardiac cycles to ensure periodicity and converged solutions^39^. Time step size was 0.2 ms with residuals set to 10^–4^ as a convergence criterion. Visualization of results including velocity patterns and wall tension was performed using ParaView (Kitware, Clifton Park, NY). Wall tension is thought to be one of the main stimuli for the dynamic response of the aorta to thicken wall stress to preferred local homeostatic level.^12^ Normalized wall tension was therefore calculated by multiplying spatial BP times the instantaneous displacement experienced beyond that during diastole.

## Results

Table 1 shows CO assessed by PC-MRI in the final week, i.e. TP-4, and estimated at younger ages based on body weight. PP obtained by catheterization prior to harvest is also provided in Table 1. Approximate human equivalent ages for the time points studied in rabbits include manifestation of mechanical stimuli from CoA around 7 years old, TP-1 at approximately 7.5 years old, TP-2 at approximately 8 years old, TP-3 at approximately 12 years old, and TP-4 around 16–17 years old.

**Table 1:** Temporal evolution of hemodynamic characteristics obtained longitudinally in a rabbit model. Cardiac output (CO) from each experimental group was quantified by 2D PC-MRI in the ascending aorta. Measured mean blood pressure (MBP) and pulsatile pressure (PP) from the catheter at final week (TP-4) and estimated at younger ages based on obstruction level % (TP-1, 2, and 3). Additional details related to branch blood flow distributions and pressures are provided in the Supplemental Materials.

Group	TP-1				TP-2				TP-3				TP-4			
	CO (ml/s)	BP (mmHg)			CO (ml/s)	BP (mmHg)		Obstr %	CO (ml/s)	BP (mmHg)		Obstr %	CO (ml/s)	BP (mmHg)		Obstr %
		MBP	PP	Obstr %		MBP	PP			MBP	PP			MBP	PP	
Control	2.13	55.7	14.9	0	2.58	55.7	14.9	0	4.76	55.7	14.9	0	6.18	55.7	14.9	0
CoA ≤ 10	2.20	71.5	17.0	69	2.53	71.5	17.2	71	3.97	71.5	18.3	76	5.23	71.5	19.5	80
CoA10-20	1.91	66.7	16.5	63	2.25	66.7	16.9	68	3.98	66.7	22.2	85	5.98	66.7	24.3	87
CoA ≥ 20	2.57	70.2	24.2	87	2.87	70.2	24.9	88	4.64	70.2	28.2	90	6.21	70.2	30.2	92
dCoA ≤ 10	2.47	63.3	16.9	68	2.78	63.3	16.8	67	4.73	63.3	15.9	45	7.02	63.3	15.6	34
dCoA10-20	1.61	65.2	18.8	78	2.07	65.2	18.5	77	4.29	65.2	16.0	50	6.64	65.2	15.7	40
dCoA ≥ 20	2.55	66.4	26.4	89	2.90	66.4	28.8	91	5.00	66.4	20.6	82	6.96	66.4	15.9	47
RdCoA ≤ 10	3.60	60.4	16.0	51	4.14	60.4	15.8	45	6.94	60.4	15.3	19	8.95	60.4	15.0	5
RdCoA10-20	2.44	63.8	16.8	66	3.05	63.8	15.8	44	5.93	63.8	15.3	20	8.19	63.8	15.3	19
RdCoA ≥ 20	2.95	56.9	21.0	83	3.46	56.9	21.8	84	6.32	56.9	15.6	33	8.46	57.0	15.6	33

 A strong correlation between PP and percent area obstruction was observed at the final TP when CoA was present. Treated conditions, i.e. dCoA and RdCoA, resulted in decreased PP as the Vicryl suture dissolved, presumably due to enlargement of the narrowing along with a corresponding decrease in coarctation severity and reflected waves. PP at younger ages was estimated based on a linear regression model of obstruction levels measured at TP-4 (Table 1).

### Wall thickness

Table 2 provides longitudinal quantification of wall thickness obtained from ultrasound images at regions proximal and distal to the CoA. Results indicate the presence of coarctation leads to arterial thickening, beyond the natural growth of the wall, proximal to the coarctation and thinning distally. The level of severity has an influence on arterial thickening proximally. For instance, the percent obstruction in CoA rabbits (e.g. CoA 10–20 mmHg BPG_pp_) increases with each time point, as does the thickness in Table 2. The duration of CoA also plays a key role in arterial thickening. Both treated CoA groups tended toward greater arterial thickness at proximal locations when the coarctation suture was present, similar to the permanent CoA groups. Operator dependence analysis showed < 13% differences among intra-observer measurements. The ICC index also revealed intra-observer agreement with mean 0.89 and 95% confidence interval [0.76, 0.98], which is considered excellent reliability according to Cicchetti et al.^40^ and good to excellent according to Koo et al.^41^.

**Table 2:** Temporal changes in thickness at proximal and distal aortic locations for representative rabbits in each group. Ultrasound sessions were processed to quantify temporal variation in thickness. Values are in millimeters.

Group	Proximal				Distal			
	TP-1	TP-2	TP-3	TP-4	TP-1	TP-2	TP-3	TP-4
Control	0.29	0.31	0.33	0.35	N/A	N/A	N/A	N/A
CoA ≤ 10	0.33	0.34	0.35	0.37	0.34	0.34	0.34	0.34
CoA10-20	0.29	0.32	0.34	0.35	0.32	0.32	0.32	0.32
CoA ≥ 20	0.32	0.33	0.37	0.40	0.34	0.33	0.30	0.28
dCoA ≤ 10	0.35	0.36	0.38	0.39	0.33	0.32	0.29	0.28
dCoA10-20	0.31	0.33	0.39	0.43	0.38	0.37	0.33	0.28
dCoA ≥ 20	0.29	0.30	0.32	0.34	0.32	0.31	0.29	0.28
RdCoA ≤ 10	0.31	0.32	0.33	0.33	0.34	0.33	0.31	0.30
RdCoA10-20	0.31	0.32	0.33	0.34	0.35	0.34	0.33	0.32
RdCoA ≥ 20	0.31	0.32	0.36	0.38	0.33	0.34	0.34	0.34

### Radial strain

Figure 2 provides example results of temporal changes in CoA shape at four time points for dissolvable CoA with a severity of ≥ 20 mmHg. Figure 2a shows diameter variation with age at three selected locations (proximal, CoA, and distal), during the diastolic phase (solid black line) that was used to develop geometries, and the systolic phase (dotted blue line) used to determine arterial wall deformation for including elastic moduli in FSI simulations. Corresponding models for each TP are shown in Fig. 2b for a representative group with corresponding local CoA modifications shown in Fig. 2c. Imaged-based creation of computational geometries was similarly conducted for all other groups. A complete summary of aortic deformation at the four TPs is presented in Table 3. The results in the control group suggest radial strain remains consistent with the ages studied (i.e. ~ 11%). In all CoA groups, radial strain in the proximal region above the coarctation was greater than was seen in control measurements. Conversely, less deformation was seen distal to the coarctation as the severity and associated pressure gradient across the coarctation increased. The proximal region of dissolvable CoA groups appeared to be exposed to smaller deformation with age as compared to permanent CoA. Radial strain variation in the final week of dissolvable CoA groups was similar between proximal and distal regions, but more pronounced in the rapid dissolvable groups, likely because the suture dissolved in the first few weeks resulting in the recovery of the local cross-sectional area at least partially, as well as any associated changes in material properties resulting from the restoration of mechanical stimuli closer to control levels.

**Table 3:** Temporal changes in the radial strain at proximal, CoA, and distal locations for a representative rabbit in each group. Images during the diastolic and systolic phases from all available ultrasound sessions were processed to quantify radial strain. Values are as percentages.

Group	Proximal				CoA				Distal			
	TP-1	TP-2	TP-3	TP-4	TP-1	TP-2	TP-3	TP-4	TP-1	TP-2	TP-3	TP-4
Control	11	11	12	12	N/A	N/A	N/A	N/A	11	11	12	12
CoA ≤ 10	10	10	11	11	0	0	0	0	11	10	9	7
CoA10-20	13	12	10	10	0	0	0	0	10	9	5	4
CoA ≥ 20	12	12	12	10	0	0	0	0	5	5	5	6
dCoA ≤ 10	14	14	10	9	0	0	13	13	13	13	11	10
dCoA10-20	10	10	9	8	0	0	12	12	8	8	8	7
dCoA ≥ 20	15	14	11	11	0	0	12	14	4	4	4	3
RdCoA ≤ 10	11	9	8	8	0	4	7	8	8	8	7	7
RdCoA10-20	11	10	9	8	0	8	11	11	8	9	8	8
RdCoA ≥ 20	10	10	8	7	0	4	6	5	4	5	6	6

### Elastic moduli

Table 4 outlines the elastic moduli proximal and distal to CoA for all time points based on material properties quantified at the end of the protocol duration i.e. TP-4. At the other TPs, elastic moduli were tuned iteratively to meet the target diameter and radial strain obtained from ultrasound images as provided in Table 4. The control group showed mild changes in elastic modulus with age at the descending aorta. Conversely, the presence of coarctation was associated with moduli reflective of arterial stiffening proximal to the coarctation and softening distal to coarctation with age. Results revealed that the level of severity correlates with increases in elastic moduli. The most severe case in permanent CoA showed prominent increases in the elastic modulus with age proximal to the CoA as compared to distally (Table 2).

**Table 4:** Temporal evolution of elastic modulus proximal and distal to the coarctation. Arterial wall properties were empirically quantified at TP-4 and estimated for younger ages during the tuning process of FSI simulations. All values are reported as kPa.

Group	Proximal				Distal			
	TP-1	TP-2	TP-3	TP-4	TP-1	TP-2	TP-3	TP-4
Control	190	200	220	220	N/A	N/A	N/A	N/A
CoA ≤ 10	130	140	180	180	130	130	100	100
CoA10-20	140	150	220	260	120	120	110	90
CoA ≥ 20	230	260	310	350	220	160	150	90
dCoA ≤ 10	290	300	330	480	250	260	320	450
dCoA10-20	200	210	340	420	200	200	340	420
dCoA ≥ 20	230	260	280	290	170	180	180	210
RdCoA ≤ 10	290	370	460	540	290	360	450	540
RdCoA10-20	150	230	260	270	150	220	240	240
RdCoA ≥ 20	280	280	350	400	280	280	350	400

### Blood flow velocity patterns

**Figure 3:**
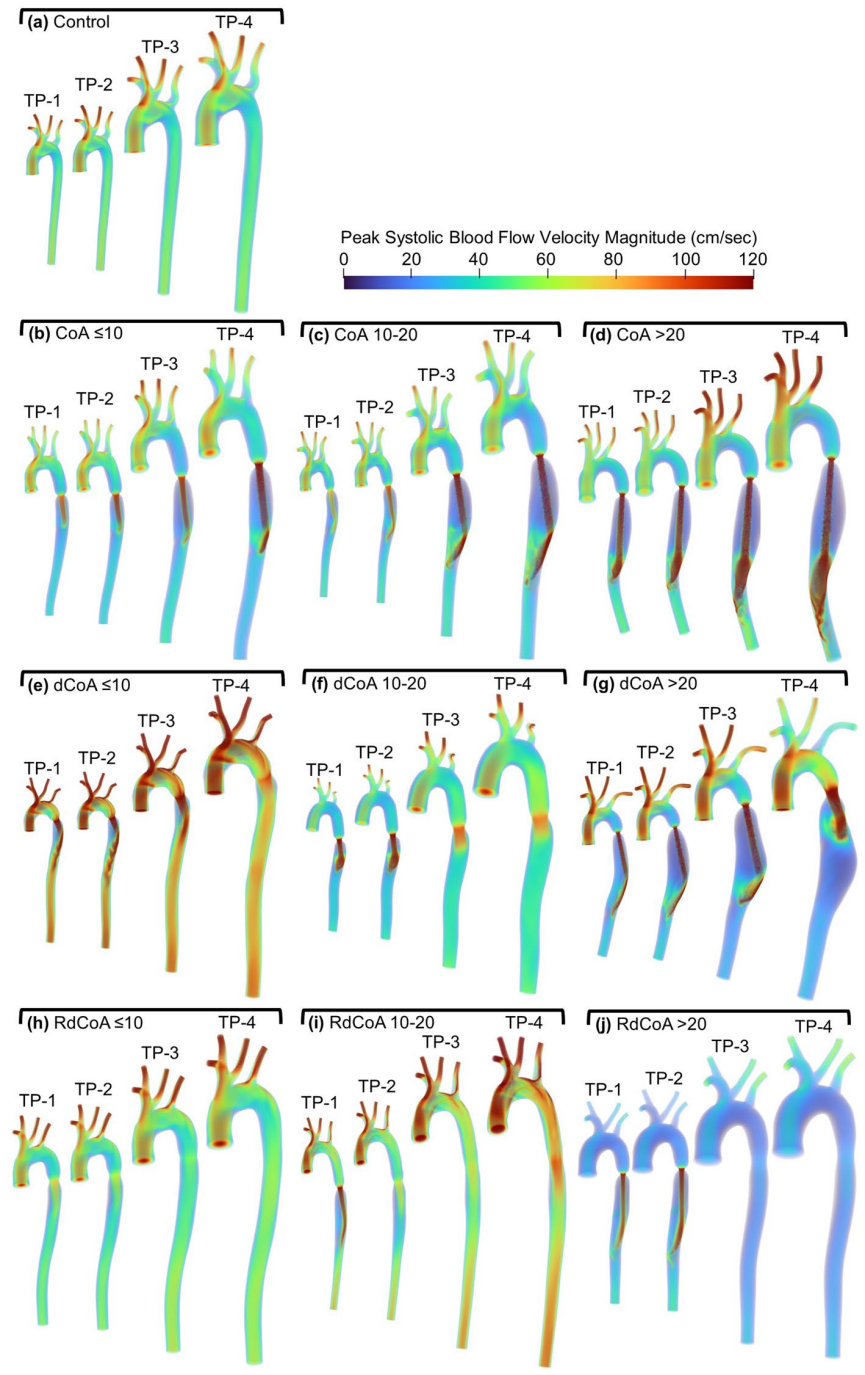
Volume rendered peak systolic blood flow velocity magnitude at four time points for a representative rabbit in each group. Results are shown for the Control group (**a**; top) as compared to permanent coarctation of the aorta (CoA; first row) with severities of CoA ≤ 10 mmHg BPG_pp_ (**b**), CoA 10–20 mmHg BPG_pp_ (**c**), and CoA ≥ 20 mmHg BPG_pp_ (**d**); Dissolvable CoA (dCoA; second row) with severities of dCoA ≤ 10 mmHg BPG_pp_ (**e**), dCoA 10–20 mmHg BPG_pp_ (**f**), dCoA ≥ 20 mmHg BPG_pp_ (**g**); and Rapid dissolvable (RdCoA; bottom row) with severities of RdCoA ≤ 10 mmHg BPG_pp_ (h), RdCoA 10–20 mmHg BPG_pp_ (**i**), RdCoA ≥ 20 mmHg BPG_pp_ (**j**). The time points shown for each group represent ages at ~ 1 (TP-1), 3 (TP-2), 10 (TP-3), and 20 weeks (TP-4) after surgery.

Simulation results for peak blood flow velocity magnitude are displayed for rabbits from each experimental group in Fig. 3. Figure 3a shows patterns in the control group that are similar for each time point in this group. However, the same findings are not observed in the CoA groups, as the severity and duration of coarctations had a dramatic impact on hemodynamics distal to the coarctation. Figure 3b-d shows blood flow velocity patterns from untreated CoA of varying severities. The velocity jet due to the narrowing becomes more severe as the coarctation severity increases, which leads to a region of post-stenotic dilation distal to the CoA. Figure 3e-j represents the treated condition of CoA resulting in morphology similar to surgical repair via resection with end-to-end anastomosis. Velocity patterns for rabbits in the dCoA group no longer show a jet from the coarctation at TP-4. Velocity results from RdCoA rabbits have restoration of general velocity patterns by TP-2 or TP-3, depending on coarctation severity (Fig. 3h-j).

### Wall tension

**Figure 4:**
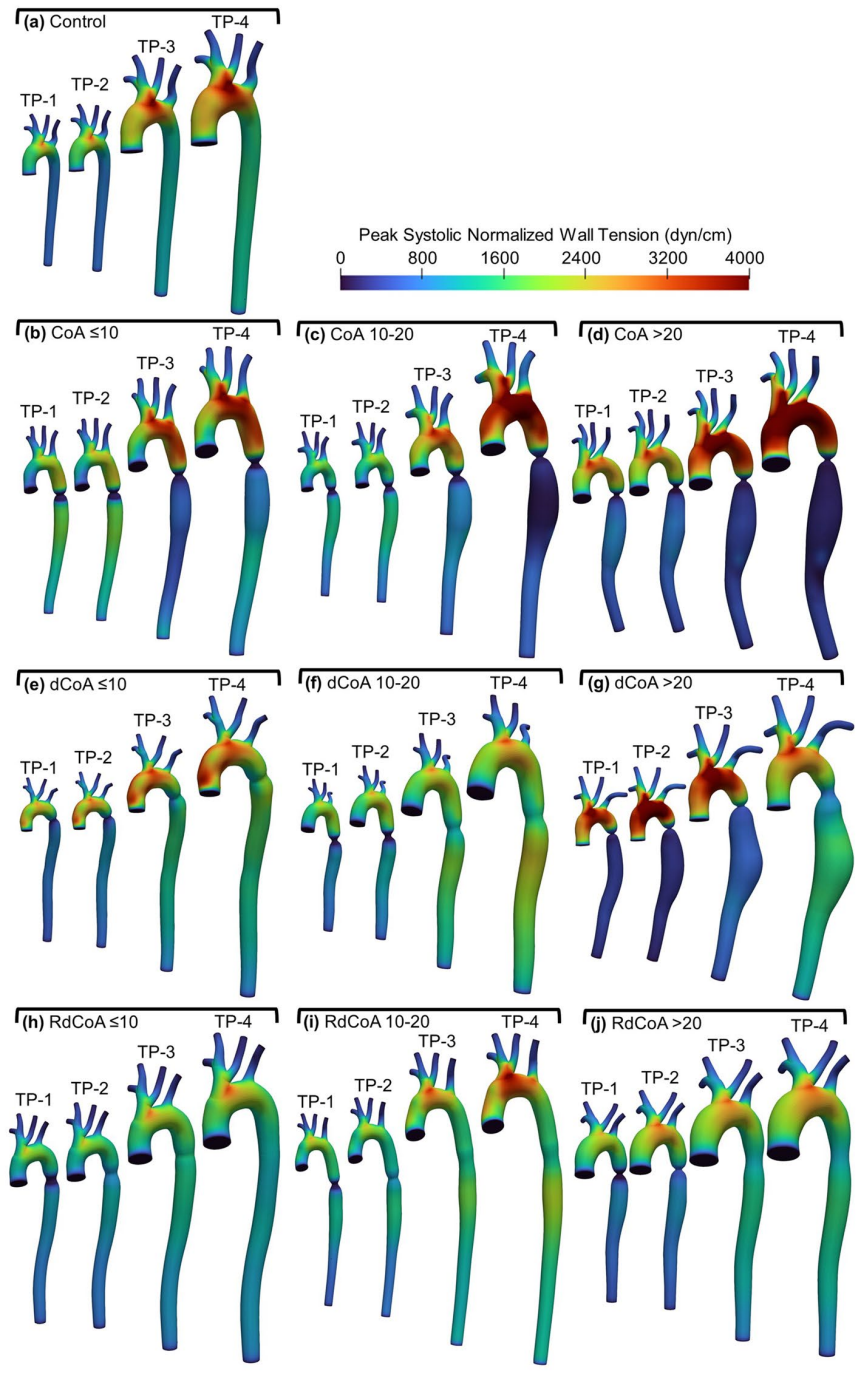
Peak systolic normalized wall tension at four time points for a representative rabbit in each group. Results are shown for the Control group (**a**; top) as compared to permanent coarctation of the aorta (CoA; first row) with severities of CoA ≤ 10 mmHg BPG_pp_ (**b**), CoA 10–20 mmHg BPG_pp_ (**c**), and CoA ≥ 20 BPG_pp_ (**d**); Dissolvable CoA (dCoA; second row) with severities of dCoA ≤ 10 mmHg BPG_pp_ (**e**), dCoA 10–20 mmHg BPG_pp_ (**f**), dCoA ≥ 20 mmHg BPG_pp_ (**g**); and Rapid dissolvable (RdCoA; bottom row) with severities of RdCoA ≤ 10 mmHg BPG_pp_ (**h**), RdCoA 10–20 mmHg BPG_pp_ (**i**), RdCoA ≥ 20 mmHg BPG_pp_ (**j**). The time points shown for each group represent ages at ~ 1 (TP-1), 3 (TP-2), 10 (TP-3), and 20 weeks (TP-4) after surgery.

Figure 4 shows FSI simulation results for a representative rabbit in each group highlighting differences in wall tension at different TPs and severities. In the control group, distributions of wall tension in the aorta generally remain consistent in their distribution with age, but with the magnitude slightly increasing from ~ 2500 (TP-1) to 4000 (TP-4) dyn/cm. All severities in the permanent CoA group showed increases in wall tension from ~ 2000 to 3000 dyn/cm at TP-1 to ~ 4000–6000 dyn/cm at TP-4, but changes were more pronounced and uniform spatially than in the control rabbit results. Arterial wall tension proximal to permanent CoA is also more prominent in the most severe case (i.e. CoA ≥ 20). In contrast, low wall tension was observed distally and corresponded with coarctation severity. Wall tension for the corrected CoA groups demonstrated a different trend with respect to age due to the opening of the coarctation region with dissolving of the associated suture. For the dCoA group, wall tension was more pronounced proximal to CoA for younger ages, i.e. TP-1 and TP-2. As the narrowing expanded due to the dissolving of the coarctation suture with age, wall tension tended to drop proximal to CoA and increase distal to CoA, i.e. TP-3 and TP-4. This is more pronounced in dCoA ≥ 20 mmHg BPG_pp_. This group demonstrated a dramatically elevated wall tension proximal to CoA at TP-2, where an obstruction level of 91% was present. This led to wall tension of ~ 6000 dyn/cm at TP-2. For comparison, the obstruction level reached 47% at TP-4 when the suture was completely dissolved. The shorter duration of coarctation in RdCoA groups demonstrated a similar trend to the control group for wall tension distributions. Since the suture dissolved after the surgery, temporal changes in wall tension remain consistent with the observation from the control group.

## Discussion

Prior studies of pathologic aortic remodeling have not yet studied the temporal evolution of mechanical stimuli at younger ages that may contribute to HTN in CoA^1,6,9,10^. For example, in prior work from our lab, Menon et al.^6^ replicated untreated and corrected CoA resulting in BPG_pp_ ≥ 20 mmHg, but was limited to the adult age (i.e. TP-4) and most severe condition studied here. The prior study by Menon et al. observed persistent endothelial dysfunction despite the correction of aortic coarctation^6^. However, specific ranges of mechanical stimuli from the severity and duration (i.e. time to treatment relative to the manifestation of mechanical stimuli from the coarctation) of CoA that could prevent such arterial remodeling were not provided.

Investigation into the temporal evolution of coarctation in human patients is challenging due to the relatively low number of heterogeneous patients at each center. Therefore, a novel rabbit model was used that served as a clinically representative model of CoA in humans^6,10^. Using this model, the severity and duration of CoA were systematically varied using permanent, dissolvable, and rapidly dissolvable sutures. A methodology was also developed to create 3D subject-specific computational geometries of the aorta from longitudinal ultrasound and MRI. Use of these geometries in FSI simulations allowed us to characterize mechanical stimuli including blood flow velocity patterns, wall tension, and radial strain indices in rabbits exposed to BPG_pp_ severities ≤ 10, 10–20, and ≥ 20 mmHg for 1, 3, or ~ 20 weeks, respectively.

The current results of blood flow patterns at TP-4 are consistent with previous observations using MRI^42,43^ and computational modeling^1,6,10,44^. General blood flow patterns and the velocity jet due to coarctation reported by Menon et al. are similar to those shown in the current study^6,10^ (i.e. CoA and dCoA ≥ 20 mmHg). Moreover, observations from human CoA patients are consistent with simulation results in our study^1,44^, where the severity of CoA was shown to impact velocity patterns downstream in native and treated cases. This suggests that the findings from our rabbit model regarding the impact of coarctation severity and duration on velocity patterns may be applicable to human CoA patients.

The temporal evolution of the descending thoracic aorta in response to coarctations of different severities and durations was computationally determined here to investigate hemodynamics at younger ages. The aortic geometry and CO were assumed to be proportional to body weight size^33,34^, resulting in a consistent Reynolds number at the ascending aorta for all time points. Peak systolic velocity magnitude in the control group with ~ 100 cm/s appeared from the ascending aorta to the common carotid and subclavian arteries. A major contribution to the variation in hemodynamics was the presence of coarctation. In permanent CoA, the area of stenosis remains the same temporally for a given severity implemented. As the severity increases, the diameter of the stenosis drops, which leads to an excessive velocity jet and a region of post-stenotic dilation distal to the coarctation. When studying velocity patterns spatially, CoA groups demonstrated lower velocity magnitude in the region proximal to CoA, in which the velocity decreases with the level of severity. This indicates that the level of severity has a marked impact on mechanical stimuli and consequently arterial remodeling, as was observed from the elastic moduli reported in Table 3.

In addition to the severity, the duration of CoA plays an important role in blood flow characteristics in the aorta. Since the rapid Vicryl suture dissolves after ~ 1 week, blood flow patterns for all severities of RdCoA were close to the control group with mild narrowing present at the location of the suture. The short duration of CoA in RdCoA groups provided temporally similar blood flow patterns even in the most severe case shown in Fig. 3j.

Wall tension was also studied for the range of CoA severities and durations seen clinically as provided during peak systole in Fig. 4. Temporal changes in wall tension in the control group indicated an increase in tension from ~ 2500 to some small regions of 4000 (TP-4) dyn/cm with age proximally, and ~ 1500–2000 dyn/cm in the descending aorta. The presence of a coarctation contributed substantially to increased wall tension and the region of the aortic arch exposed to it. Observations from the untreated CoA groups showed that as the obstruction level increases, wall tension tends to increase proximal and decrease distal to the coarctation. The most severe case in permanent CoA demonstrated the highest wall tension in excess of 4000 dyn/cm throughout most of the proximal aorta in adulthood (i.e. TP-4). Conversely, the treated CoA showed a drop in the wall tension during adulthood but the presence of stenosis at early ages caused pronounced wall tension proximal to CoA. This is more pronounced in dCoA ≥ 20 mmHg in which wall tension reached ~ 4000 dyn/cm and dropped to ~ 2300 dyn/cm in TP-2 and TP-4, respectively. Observations from simulations involving the shortest duration of CoA (i.e. RdCoA) indicated a mild impact on wall tension and consequently less arterial remodeling as suggested by the temporal changes in stiffening and thickening reported in Tables 2 and 4, respectively.

The simulated wall tension values from the control group at TP-4 (Fig. 4a) are aligned with the estimated values reported by Wolinsky et al.^12^ (i.e., ~ 1900 dyn/cm in the thoracic aorta). Wolinsky et al. also indicated wall tension for small animals increased by ~ 5000 dyn/cm for every 1 kg in body weight, but only 500 dyn/cm for every kg in large animals. They defined small animals as those weighing ≤ 2 kg, while large animals were defined as those ≥ 5 kg. Using this designation, our rabbits began the current study as small animals but were closer to the prior designation of larger animals by TP-4. The temporal changes in wall tension proximal to CoA in our rabbit model are generally aligned with the prior findings from Wolinsky et al. in this regard. For instance, wall tension in the control group increased ~ 1000 dyn/cm for an ~ 2.0 kg increase in body weight size from TP-1 to TP-4. However, for CoA groups, the presence of coarctation magnified the wall tension. The prominent example in the CoA groups is the severe case in permanent CoA (i.e. CoA ≥ 20 mmHg BPG_pp_) in which wall tension proximal to CoA approximately doubled, increasing by ~ 2000 dyn/cm, corresponding to ~ 2 kg rise in body weight size. Importantly, these results indicate stimuli for remodeling from wall tension in the proximal aorta can exceed values seen in adulthood under control conditions, and much earlier (e.g. dCoA ≤ 10 mmHg BPG_pp_). In other words, it appears that even mild CoA has the potential to introduce stimuli for remodeling that exceeds those seen in adulthood if not treated early and for a BPG_pp_ severity threshold much lower than that currently seen in the treatment guidelines for CoA^4,5^.

The mechanical stimuli induced by the severity and duration of CoA seem to correlate with arterial thickening and stiffening results (Table 2). Arterial thickening was previously reported for human patients^45–47^ and animal models^48–51^ exposed to untreated and treated coarctation conditions. Arterial thickening and stiffening were also quantified in response to untreated and treated CoA. Thickness measurements were generally consistent in corrected rabbits with wall tension results similar to those seen in control (i.e. dCoA ≤ 10 mmHg BPG_pp_). A shorter duration of coarctation (RdCoA, ~ 1wk) also showed similar thickness results to the control group. In contrast, a longer CoA duration (dCoA, ~ 4wks) led to arterial remodeling, which is more pronounced in the most severe case where wall tension was pronounced in early young ages (see Fig. 4g). These observations suggest that in addition to CoA severity, the duration of coarctation is an important factor for the stimuli influencing the remodeling of the proximal aorta. CoA present for a shorter duration seems to have a minor impact on the arterial wall alternations proximal and distal to the CoA.

**Figure 5:**
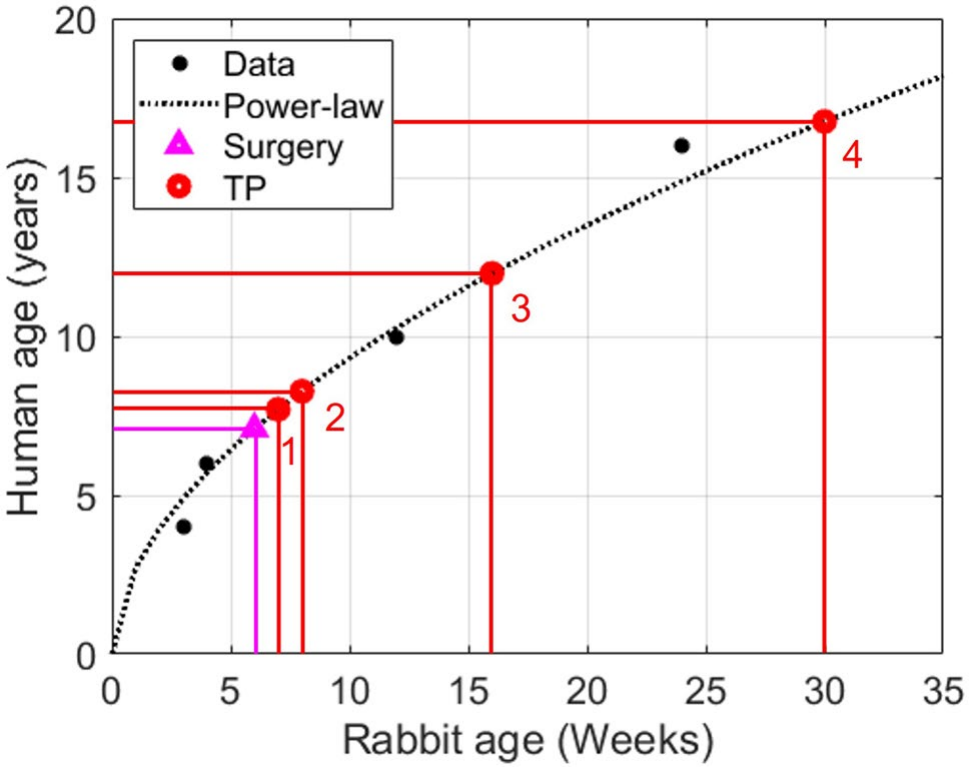
The relationship between human and rabbit ages as reported in the literature. Data were fit to the allometric (power-law) equation, which is wildly used for scaling in biology. Approximate human equivalent ages for the time points studied in rabbits include manifestation of mechanical stimuli from CoA around 7 years old, TP-1 at approximately 7.5 years old, TP-2 at approximately 8 years old, TP-3 at approximately 12 years old, and TP-4 around 16–17 years old.

Wolinsky et al. also showed that the wall tension was proportional to the body weight size across different species, and hence the average tension per lamellar unit of aortic media was strikingly similar and independent of species^12^. The aorta seems to remodel and thicken in response to the tension presented. The lifespan is undoubtedly different between rabbits and humans. Figure 5 provides guidance relating time points from the current study to the human lifespan^52^. The collective application of these findings suggests our results may be applicable to other species (i.e. humans with CoA) according to body size and may provide guidance for values of the mechanical stimuli that could be used to predict the amount of wall thickening and stiffening expected in response to CoA in human patients with CoA.

The current results should be interpreted relative to several potential limitations. FSI simulations were limited to one representative rabbit model per group. Future work requires consideration of a larger sample size to confirm the current findings. FSI simulations were performed using empirical data (those from experimental measurements) obtained in the final week (TP-4). However, the computational geometries and boundary conditions for younger ages were estimated based on longitudinal ultrasound images, body weight, and data at TP-4. There may be differences in the way the current work calculated wall tension as compared to that of prior work (e.g. Wolinsky et al.^12^), but the conclusions presented above are not likely to be substantially altered by such differences. CO at younger ages was estimated based on the body weight as previously reported for healthy rabbits^33,34^. Since there is no report for diseased rabbits, a similar assumption was made for CoA groups. Linear material properties were assumed for the arterial wall as implemented in the numerical formulation used for FSI (i.e. coupled momentum method)^31^.

Blood pressures from rabbit data used in the current study are lower than would be expected since measurements were obtained during Isoflurane at ~ 1 MAC. However, a recent study from our group using computational methods to implement simulated conscious hemodynamic conditions using FSI did not show significant changes in velocity and pressure gradients^21^.

Important geometric features in CoA include the length and diameter of the coarctation segment. CoA shape in the current study represents a short discrete stenosis that did not change for the untreated coarctation groups over time. Distal dilation beyond the coarctation was observed in the current study and reported. However, a longer coarctation length may lead to different conclusions and therefore requires further study. Data for the representative rabbit models of each group featured male rabbits for consistency and because of a slight propensity for CoA to present more frequently in males^53^.

Distributions of wall shear stress are not reported in the current work for several reasons. First, we generally ascribe to the notion of O'Rourke and Cartmill^54^ who suggested most of the morbidity in CoA can be explained on the basis of altered conduit (blood flow) and cushioning (capacitance) functions. The authors described how CoA introduces a BP wave reflection site near the heart causing drastic reductions in aortic capacitance and elevated pulse pressure. These findings are consistent with hypertension often observed in CoA patients. Concomitant increases in afterload also offer an explanation for heart failure that can occur if CoA goes untreated. Importantly, mechanical stimuli associated with these forms of morbidity are primarily occurring above the site of the coarctation. We previously studied wall shear stress proximally and observed only modest differences between different groups^6,10^. This prior work used the maximum untreated and treated BPG_pp_ groups (i.e. CoA and dCoA > 20 mmHg) that are extended upon in the current work. Hence, wall shear stress results are not likely to differ for the current study using more refined, but lower BPG_pp_ levels.

The current study presents the temporal evolution of mechanical stimuli with age using results from an experimental rabbit model integrated within a framework for FSI simulations using subject-specific models for the range of CoA severities and durations seen clinically. The indices reported for these groups were discussed relative to the knowledge that excessive coarctation-induced mechanical stimuli over time can lead to irreversible arterial remodeling and dysfunction. The results indicate that besides the level of severity, duration of coarctation also plays an important role in arterial remodeling and provides a range of values for mechanical stimuli implicated in morbidity that could be derived from remodeling proximal to the coarctation. Future work will extend the current results to further predict the range of temporal mechanical stimuli presented here that can avoid permanent vascular remodeling in response to CoA in humans.

## Supplementary Information

Supplementary Information.

## Data Availability

The datasets used and/or analyzed during the current study are included in this published article and available from the corresponding author on reasonable request.
