# Supplementary material for: Temporal evolution of mechanical stimuli from vascular remodeling in response to the severity and duration of aortic coarctation in a preclinical model
Source: Sci Rep. 2023 May 23;13:8352. doi: 10.1038/s41598-023-34400-8 (PMC10205817; doi:10.1038/s41598-023-34400-8)
Supplement: Supplementary file 1 — Supplementary Information. [file 41598_2023_34400_MOESM1_ESM.pdf]

Temporal evolution of mechanical stimuli from vascular remodeling in response to the severity and duration of aortic coarctation in a preclinical model

Supplemental Materials

Authors:

Jamasp Azarnoosh Ph.D. [1,2]*, Arash Ghorbannia Ph.D. [1,6], El-Sayed H. Ibrahim Ph.D. [3], Hilda Jurkiewicz B.S. [1,2], Lindsey Kalvin B.S.[4], John F. LaDisa Jr. Ph.D.[1,2,4,5,6]

Affiliations:

[1]Department of Pediatrics - Section of Cardiology, Medical College of Wisconsin, Milwaukee, WI, USA

[2]Department of Biomedical Engineering, Marquette University and the Medical College of Wisconsin, Milwaukee, WI, USA

Departments of [3]Radiology, [4]Medicine - Division of Cardiovascular Medicine, and [5]Physiology, Medical College of Wisconsin, Milwaukee, WI, USA

[6]Herma Heart Institute, Children's Wisconsin, Milwaukee, WI, USA

Corresponding Author:

Jamasp Azarnoosh Ph.D.
jazarnoosh@mcw.edu
423-314-9280

Blood flow from 2D Phase-contrast MRI (PC-MRI) in the ascending was measured to generate inflow waveforms for TP-4 and estimated at younger ages (TP-1,2, and 3) based on body weight as shown in Figure S1.

Cardiac output was obtained from PC-MRI in the ascending aorta as provided in Table 1. Table S1 provides temporal evolution of hemodynamic characteristics obtained longitudinally at each branch as an extension of Table 1. Table S1 summarizes cardiac output, mean blood pressure (MBP), and pulsatile pressure (PP) at outlets (right subclavian artery (RS), right common carotid (RC), left common carotid (LC), left subclavian artery (LS), and distal artery (DA)).

Three-element Windkessel parameters are estimated using total resistance and time constants as described in the body of the manuscript. Table S2 provides calculated RCR parameters at the four time points of all groups. Table S3 summarizes RCR parameters and time constant (τ) values at each outlet.

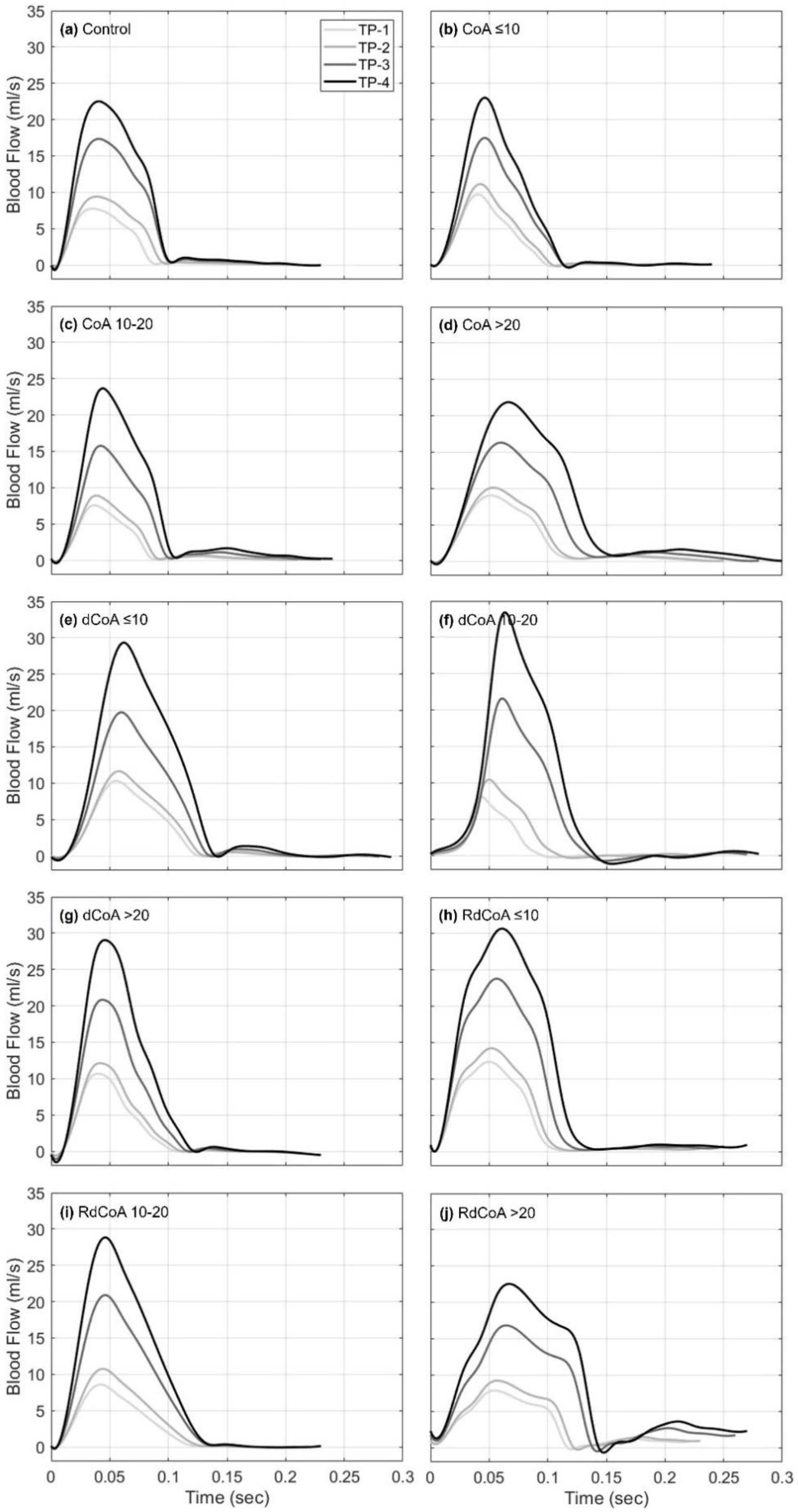

Figure S1. Measured blood flow from 2D PC-MRI in the ascending aorta to generate inflow waveforms for TP-4. Inflow waveforms at younger ages (TP-1,2, and 3) are estimated based on body weight.

Table S1. Measured flow rate from 2D PC-MRI, mean blood pressure (MBP), and pulsatile pressure (PP) from catheterization at the final time point (TP-4) and estimated at younger ages (TP-1,2, and 3). Values represent those at outlets i.e. right subclavian artery (RS), right common carotid (RC), left common carotid (LC), left subclavian artery (LS), and distal artery (DA). Flow rate and pressure units are ml/s and mmHg, respectively.

		TP-1					TP-2					TP-3					TP-4				
Group		RS	RC	LC	LS	DA	RS	RC	LC	LS	DA	RS	RC	LC	LS	DA	RS	RC	LC	LS	DA
Control	Flow rate	0.18	0.15	0.13	0.18	1.49	0.22	0.18	0.15	0.22	1.81	0.40	0.33	0.28	0.40	3.34	0.52	0.43	0.37	0.52	4.34
	MBP	55.7	55.7	55.7	55.7	54.0	55.7	55.7	55.7	55.7	54.0	55.7	55.7	55.7	55.7	54.0	55.7	55.7	55.7	55.7	54.0
	PP	14.9	14.9	14.9	14.9	21.3	15.7	13.3	13.2	14.9	19.6	15.5	14.9	14.9	15.4	20.1	16.0	15.3	14.5	14.7	19.4
CoA≤10	Flow rate	0.21	0.13	0.16	0.21	1.48	0.24	0.15	0.19	0.24	1.70	0.38	0.24	0.29	0.38	2.67	0.51	0.32	0.38	0.50	3.52
	MBP	71.5	71.5	71.5	71.5	67.5	71.5	71.5	71.5	71.5	67.5	71.5	71.5	71.5	71.5	67.5	71.5	71.5	71.5	71.5	67.5
	PP	17.1	17.1	17.1	17.1	22.1	17.2	17.2	17.2	17.2	21.8	18.4	18.4	18.4	18.4	19.5	19.5	19.5	19.5	19.5	17.5
CoA10-20	Flow rate	0.13	0.18	0.15	0.12	1.33	0.15	0.21	0.18	0.15	1.57	0.26	0.37	0.32	0.26	2.78	0.39	0.55	0.48	0.39	4.17
	MBP	66.7	66.7	66.7	66.7	56.8	66.7	66.7	66.7	66.7	56.8	66.7	66.7	66.7	66.7	56.8	66.7	66.7	66.7	66.7	56.8
	PP	16.5	16.5	16.5	16.5	23.3	16.9	16.9	16.9	16.9	22.5	22.2	22.2	22.2	22.2	13.6	24.3	24.3	24.3	24.3	11.0
CoA≥20	Flow rate	0.27	0.21	0.20	0.27	1.62	0.30	0.23	0.22	0.30	1.81	0.49	0.38	0.36	0.49	2.92	0.66	0.50	0.49	0.66	3.91
	MBP	70.2	70.2	70.2	70.2	51.5	70.2	70.2	70.2	70.2	51.5	70.2	70.2	70.2	70.2	51.5	70.2	70.2	70.2	70.2	51.5
	PP	24.2	24.2	24.2	24.2	11.1	24.9	24.9	24.9	24.9	10.2	28.2	28.2	28.2	28.2	6.7	30.2	30.2	30.2	30.2	4.7
dCoA≤10	Flow rate	0.19	0.19	0.19	0.19	1.71	0.22	0.21	0.21	0.22	1.93	0.37	0.36	0.36	0.37	3.27	0.55	0.53	0.54	0.55	4.86
	MBP	63.4	63.4	63.4	63.4	61.7	63.4	63.4	63.4	63.4	61.7	63.4	63.4	63.4	63.4	61.7	63.4	63.4	63.4	63.4	61.7
	PP	16.9	16.9	16.9	16.9	22.4	16.8	16.8	16.8	16.8	22.7	15.9	15.9	15.9	15.9	24.0	15.6	15.6	15.6	15.6	23.5
dCoA10-20	Flow rate	0.22	0.17	0.21	0.23	1.72	0.26	0.20	0.24	0.26	1.96	0.44	0.34	0.41	0.44	3.37	0.61	0.47	0.58	0.61	4.69
	MBP	66.4	66.4	66.4	66.4	63.8	66.4	66.4	66.4	66.4	63.8	66.4	66.4	66.4	66.4	63.8	66.4	66.4	66.4	66.4	63.8
	PP	26.4	26.4	26.4	26.4	8.6	28.8	28.8	28.8	28.8	6.1	20.6	20.6	20.6	20.6	15.8	15.9	15.9	15.9	15.9	24.0
dCoA≥20	Flow rate	0.42	0.27	0.24	0.42	2.26	0.48	0.31	0.28	0.48	2.60	0.80	0.52	0.47	0.80	4.36	1.03	0.66	0.60	1.03	5.62
	MBP	60.8	60.8	60.8	60.8	60.6	60.8	60.8	60.8	60.8	60.6	60.8	60.8	60.8	60.8	60.6	60.4	60.4	60.4	60.4	59.9
	PP	16.0	16.0	16.0	16.0	24.0	15.8	15.8	15.8	15.8	23.9	15.3	15.3	15.3	15.3	22.6	15.0	15.0	15.0	15.0	21.7
RdCoA≤10	Flow rate	0.18	0.18	0.20	0.18	1.70	0.23	0.23	0.25	0.23	2.12	0.44	0.44	0.49	0.44	4.12	0.61	0.61	0.68	0.60	5.69
	MBP	62.4	62.4	62.4	62.4	60.2	62.4	62.4	62.4	62.4	60.2	62.4	62.4	62.4	62.4	60.2	63.8	63.8	63.8	63.8	61.5
	PP	16.8	16.8	16.8	16.8	22.7	15.8	15.8	15.8	15.8	23.9	15.3	15.3	15.3	15.3	22.6	15.3	15.3	15.3	15.3	22.5
RdCoA10-20	Flow rate	0.18	0.18	0.20	0.18	1.70	0.23	0.23	0.25	0.23	2.12	0.44	0.44	0.49	0.44	4.12	0.61	0.61	0.68	0.60	5.69
	MBP	62.4	62.4	62.4	62.4	60.2	62.4	62.4	62.4	62.4	60.2	62.4	62.4	62.4	62.4	60.2	63.8	63.8	63.8	63.8	61.5
	PP	16.8	16.8	16.8	16.8	16.8	15.8	15.8	15.8	15.8	23.9	15.3	15.3	15.3	15.3	22.6	15.3	15.3	15.3	15.3	22.5
RdCoA≥20	Flow rate	0.35	0.25	0.30	0.35	1.69	0.41	0.30	0.35	0.41	1.98	0.75	0.54	0.65	0.75	3.62	1.01	0.73	0.87	1.01	4.85
	MBP	54.7	54.7	54.7	54.7	53.5	54.7	54.7	54.7	54.7	53.5	54.7	54.7	54.7	54.7	53.5	56.9	56.9	56.9	56.9	55.5
	PP	21.0	21.0	21.0	21.0	15.3	21.8	21.8	21.8	21.8	14.1	15.6	15.6	15.6	15.6	23.5	15.6	15.6	15.6	15.6	23.4

Table S2. Windkessel RCR parameters and time constant (τ) values for the entire system at the selected time points. Values were distributed to each outlet as described in the manuscript and shown in Table S3. R_c and R_d are in units of dyn·s/cm^5, C is in units of cm^5/dyn, and τ is in units of s.

	TP-1				TP-2				TP-3				TP-4			
Group	R_c	C	R_d	τ	R_c	C	R_d	τ	R_c	C	R_d	τ	R_c	C	R_d	τ
Control	874	1.4E-04	34073	4.68	576	1.6E-04	28225	4.68	390	3.1E-04	15223	4.68	601	4.1E-04	11425	4.68
CoA≤10	1513	6.0E-05	41708	2.51	1131	7.1E-05	36561	2.60	600	1.5E-04	23388	3.50	728	2.3E-04	17480	3.96
CoA10-20	1764	4.0E-05	44663	1.80	1779	5.3E-05	37757	2.00	960	9.4E-05	21367	2.00	997	1.4E-04	13878	1.99
CoA≥20	3019	4.8E-05	40103	1.94	1943	5.7E-05	33385	1.92	959	9.1E-05	20361	1.85	903	1.3E-04	14144	1.89
dCoA≤10	1025	7.7E-05	33148	2.55	909	8.8E-05	29382	2.60	803	2.0E-04	17049	3.47	541	3.4E-04	11477	3.92
dCoA10-20	3240	4.3E-05	50757	2.21	2515	5.7E-05	39404	2.24	1114	1.6E-04	19147	3.00	785	3.3E-04	12296	4.00
dCoA≥20	1320	5.9E-05	31685	1.88	1394	1.3E-04	27649	3.50	697	2.3E-04	16729	3.80	762	3.4E-04	11933	4.00
RdCoA≤10	1118	1.5E-04	21247	3.26	972	1.9E-04	18464	3.58	638	4.7E-04	10965	5.16	450	5.4E-04	8549	4.58
RdCoA10-20	1161	1.2E-04	32022	4.00	919	2.6E-04	26927	7.00	501	4.3E-04	13815	6.00	363	5.2E-04	10009	5.22
RdCoA≥20	7308	8.8E-05	17052	1.50	14318	2.4E-04	7052	1.70	1332	4.1E-04	10251	4.20	806	5.4E-04	8154	4.40

Table S3. Windkessel RCR parameters and time constant (τ) values for each time point and outlet i.e. right subclavian artery (RS), right common carotid (RC), left common carotid (LC), left subclavian artery (LS), and distal artery (DA). R_c and R_d are in units of dyn·s/cm^5, C is in units of cm^5/dyn, and τ is in units of s.

		TP-1					TP-2					TP-3					TP-4				
Group	RCR	RS	RC	LC	LS	DA	RS	RC	LC	LS	DA	RS	RC	LC	LS	DA	RS	RC	LC	LS	DA
Control	R_c	16653	12558	14632	20804	14441	13725	8280	9648	17146	11902	7440	5611	6538	10225	8173	7164	5186	5036	7876	5964
	C	1.2E-05	9.6E-06	8.2E-06	1.2E-05	4.6E-05	1.4E-05	1.2E-05	9.9E-06	1.4E-05	5.5E-05	2.6E-05	2.1E-05	1.8E-05	2.6E-05	1.1E-04	3.4E-05	2.8E-05	2.4E-05	3.4E-05	1.4E-04
	R_d	399673	489776	570667	395282	33696	329392	405721	472730	325773	27771	178571	218829	254971	175680	13335	136112	167689	196392	135318	10602
	τ	4.68	4.68	4.68	4.68	1.53	4.68	4.68	4.68	4.68	1.53	4.68	4.68	4.68	4.68	1.53	4.68	4.68	4.68	4.68	1.53
CoA≤10	R_c	17882	21287	20594	24812	21208	15594	18564	17960	19670	18495	7444	7876	8164	10015	7735	9417	11957	9915	11403	20421
	C	5.8E-06	3.6E-06	4.4E-06	5.8E-06	8.1E-05	6.9E-06	4.3E-06	5.2E-06	6.8E-06	8.7E-05	1.4E-05	9.1E-06	1.1E-05	1.4E-05	9.7E-05	2.2E-05	1.4E-05	1.7E-05	2.2E-05	5.8E-05
	R_d	429170	688282	567819	426312	39386	374265	600227	495175	373739	34347	240675	385942	318411	240364	25896	178914	286965	237967	178644	5105
	τ	2.51	2.51	2.51	2.51	3.20	2.60	2.60	2.60	2.60	3.00	3.50	3.50	3.50	3.50	2.50	3.96	3.96	3.96	3.96	2.94
CoA10-20	R_c	12119	19540	22565	15134	43190	11467	18720	21618	15817	36780	11859	14478	16791	15546	10903	6772	10771	12492	11047	13620
	C	2.8E-06	3.8E-06	3.3E-06	2.7E-06	2.1E-04	3.7E-06	5.0E-06	4.4E-06	3.6E-06	2.5E-04	6.1E-06	8.8E-06	7.6E-06	6.1E-06	2.1E-05	9.4E-06	1.3E-05	1.1E-05	9.2E-06	8.6E-04
	R_d	661164	468972	541553	672775	14397	561888	397288	458775	569993	12260	326964	226824	263062	329920	16355	218956	149988	173951	219107	4540
	τ	1.80	1.80	1.80	1.80	2.98	2.00	2.00	2.00	2.00	3.02	2.00	2.00	2.00	2.00	3.46	1.99	1.99	1.99	1.99	3.92
CoA≥20	R_c	24833	28813	31899	33654	4735	22844	23247	24059	28698	3875	10786	11413	11809	17636	1938	8529	7421	8653	9945	1404
	C	5.8E-06	4.7E-06	4.6E-06	6.0E-06	7.1E-05	6.3E-06	4.8E-06	4.6E-06	6.4E-06	1.0E-04	9.9E-06	7.6E-06	7.4E-06	9.9E-06	2.3E-04	1.4E-05	1.1E-05	1.0E-05	1.4E-05	4.3E-04
	R_d	329928	414465	423795	320599	54450	303501	399421	413386	297417	39185	185317	242207	250606	178323	23900	133620	178093	183629	132126	16147
	τ	1.94	1.94	1.94	1.94	3.86	1.92	1.92	1.92	1.92	4.07	1.85	1.85	1.85	1.85	5.53	1.89	1.89	1.89	1.89	6.88
dCoA≤10	R_c	19565	13647	13448	19555	5776	17342	12096	11920	17333	5333	10221	7129	7025	10216	3269	6880	3999	4729	6113	2031
	C	6.0E-06	5.8E-06	5.8E-06	6.1E-06	7.1E-05	7.0E-06	6.7E-06	6.7E-06	7.0E-06	8.1E-05	1.6E-05	1.5E-05	1.6E-05	1.6E-05	1.3E-04	1.7E-05	1.6E-05	1.7E-05	1.7E-05	1.9E-04
	R_d	415205	441244	434813	415001	42355	368030	391109	385410	367848	37330	216906	230509	227150	216800	21875	146012	155968	152907	146704	14895
	τ	2.55	2.55	2.55	2.55	3.02	2.60	2.60	2.60	2.60	3.01	3.47	3.47	3.47	3.47	2.82	3.92	3.92	3.92	3.92	2.80
dCoA10-20	R_c	37163	30273	31467	55664	8865	28851	20144	20939	38412	6882	12783	8114	8434	16245	4158	9003	6286	6534	11987	2506
	C	3.8E-06	2.7E-06	2.6E-06	3.8E-06	4.8E-05	5.0E-06	3.5E-06	3.4E-06	5.0E-06	6.2E-05	1.4E-05	9.8E-06	9.4E-06	1.4E-05	1.1E-04	2.8E-05	2.0E-05	2.0E-05	2.8E-05	1.9E-04
	R_d	582215	834662	867595	562822	65012	451992	651333	677031	441738	50471	219629	316439	328925	215832	23563	141045	203250	211269	137846	15392
	τ	2.21	2.21	2.21	2.21	3.14	2.24	2.24	2.24	2.24	3.12	3.00	3.00	3.00	3.00	2.69	4.00	4.00	4.00	4.00	2.88
dCoA≥20	R_c	15336	14077	19567	22993	22993	16194	14599	19729	21245	3849	7925	6457	8423	11881	19889	8660	9408	9204	20196	14497
	C	5.1E-06	3.9E-06	4.8E-06	5.1E-06	1.1E-04	1.1E-05	8.3E-06	1.0E-05	1.1E-05	1.9E-04	2.0E-05	1.5E-05	1.9E-05	2.0E-05	6.6E-04	2.9E-05	2.2E-05	2.8E-05	3.2E-05	6.9E-04
	R_d	368070	488677	388080	360224	34911	321187	427804	338984	315971	31144	190212	251835	202155	186139	4972	135679	178753	144198	124058	3624
	τ	1.88	1.88	1.88	1.88	4.00	3.50	3.50	3.50	3.50	6.00	3.80	3.80	3.80	3.80	3.26	4.00	4.00	4.00	4.00	2.50
RdCoA≤10	R_c	9701	9031	11671	10668	8832	8430	7848	10143	8428	7675	5536	5466	6920	5534	5534	3903	3028	4025	4292	2843
	C	1.8E-05	1.1E-05	1.0E-05	1.8E-05	6.0E-05	2.2E-05	1.4E-05	1.3E-05	2.2E-05	1.0E-04	5.4E-05	3.5E-05	3.2E-05	5.4E-05	1.8E-04	6.2E-05	4.0E-05	3.6E-05	6.2E-05	2.7E-04
	R_d	184314	292006	321798	183293	26495	160171	253758	279647	160127	23025	95115	150707	166078	95089	14662	74162	118101	130153	73752	11372
	τ	3.26	3.26	3.26	3.26	1.60	3.58	3.58	3.58	3.58	2.30	5.16	5.16	5.16	5.16	2.58	4.58	4.58	4.58	4.58	3.06
RdCoA10-20	R_c	13425	15642	17998	19335	20698	9388	12376	12754	13206	17400	5792	5784	6040	6790	7554	4196	4889	4376	5622	4609
	C	9.3E-06	9.3E-06	1.0E-05	9.2E-06	5.9E-05	1.9E-05	1.9E-05	2.2E-05	1.9E-05	9.4E-05	3.2E-05	3.2E-05	3.6E-05	3.2E-05	1.9E-04	3.9E-05	3.9E-05	4.3E-05	3.8E-05	2.6E-04
	R_d	434072	431277	381967	430310	25298	366134	362661	322881	364118	21267	187273	187031	166518	187202	12326	135679	134806	120643	134925	9794
	τ	4.02	4.02	4.02	4.02	1.48	7.01	7.01	7.01	7.01	2.00	5.98	5.98	5.98	5.98	2.30	5.22	5.22	5.22	5.22	2.50
RdCoA≥20	R_c	61237	85067	71468	61251	7590	119979	166667	140023	120005	5258	11162	14157	13027	10679	10679	6757	7301	7886	7885	10669
	C	1.0E-05	7.6E-06	9.0E-06	1.0E-05	9.5E-05	2.9E-05	2.1E-05	2.5E-05	2.9E-05	1.1E-04	4.9E-05	3.5E-05	4.2E-05	4.9E-05	4.6E-04	6.4E-05	4.5E-05	5.5E-05	6.5E-05	5.2E-04
	R_d	142886	198489	166758	142918	34578	59094	82090	68967	59107	31005	85899	120674	100250	86404	5906	68323	96996	79737	67212	4573
	τ	1.50	1.50	1.50	1.50	3.3	1.70	1.70	1.70	1.70	3.4	4.20	4.20	4.20	4.20	2.70	4.40	4.40	4.40	4.40	2.40
